# A Tachyplesin Antimicrobial Peptide from Theraphosidae Spiders with Potent Antifungal Activity Against *Cryptococcus neoformans*

**DOI:** 10.3390/microorganisms12122648

**Published:** 2024-12-20

**Authors:** Brenda B. Michira, Yi Wang, James Mwangi, Kexin Wang, Demeke Asmamaw, Dawit Adisu Tadese, Jinai Gao, Mehwish Khalid, Qiu-Min Lu, Ren Lai, Juan Li

**Affiliations:** 1 Key Laboratory of Genetic Evolution & Animal Models, Engineering Laboratory of Peptides of Chinese Academy of Sciences, Key Laboratory of Bioactive Peptides of Yunnan Province, KIZ-CUHK Joint Laboratory of Bioresources and Molecular Research in Common Diseases, National Resource Center for Non-Human Primates, and Sino-African Joint Research Center, New Cornerstone Science Laboratory, Kunming Institute of Zoology, The Chinese Academy of Sciences, Kunming 650201, China; brenda@mail.kiz.ac.cn (B.B.M.); jams@mail.kiz.ac.cn (J.M.); wangkexin@cibr.ac.cn (K.W.); demeke2004@gmail.com (D.A.); davadisu@gmail.com (D.A.T.); gaojinai18@mails.ucas.ac.cn (J.G.); mehwishkhalidd120@gmail.com (M.K.); lvqm@mail.kiz.ac.cn (Q.-M.L.); 2 University of Chinese Academy of Sciences, Beijing 100049, China; 3 Kunming College of Life Science, University of Chinese Academy of Sciences, Kunming 650204, China; 4 Center for Evolution and Conservation Biology, Southern Marine Science and Engineering Guangdong Laboratory (Guangzhou), Guangzhou 511458, China; 13477288321@163.com; 5 Medical College of Tianjin University, Tianjin University, Tianjin 300072, China; 6 School of Molecular Medicine, Hangzhou Institute for Advanced Study, University of Chinese Academy of Sciences, Hangzhou 310024, China

**Keywords:** antimicrobial peptides, antifungal, membrane disruption, *Chilobrachys liboensis*, spider venom transcriptome, *Cryptococcus neoformans*, tachyplesins

## Abstract

The venoms of Theraphosidae spiders have evolved into diverse natural pharmacopeias through selective pressures. *Cryptococcus neoformans* is a global health threat that frequently causes life-threatening meningitis and fungemia, particularly in immunocompromised patients. In this study, we identify a novel anti-*C. neoformans* peptide, QS18 (QCFKVCFRKRCFTKCSRS), from the venom gland of China’s native spider species *Chilobrachys liboensis* by utilizing bioinformatic tools. QS18 shares over 50% sequence similarity with tachyplesin peptides, previously identified only in horseshoe crab hemocytes, expanding the known repertoire of the tachyplesin family to terrestrial arachnids. The oxidative folding of QS18 notably enhances its antifungal activity and stability, resulting in a minimum inhibitory concentration of 1.4 µM. The antimicrobial mechanism of QS18 involves cell membrane disruption. QS18 exhibits less than 5% hemolysis in human erythrocytes, indicating microbial selectivity and a favorable safety profile for therapeutic use. Furthermore, mouse model studies highlight QS18’s ability as an antifungal agent with notable anti-inflammatory activity. Our study demonstrates QS18 as both a promising template for spider venom peptide research and a novel candidate for the development of peptide antifungals.

## 1. Introduction

Advancements in medical treatments, such as immunosuppressive therapies and organ transplants, have inadvertently increased the incidence of opportunistic fungal infections [1]. *Cryptococcus neoformans* infection can lead to cryptococcal meningitis, a potentially fatal and prevalent systemic disease with significant global impact [2]. Approximately 223,100 cases of cryptococcal infections are reported annually worldwide, leading to over 180,000 deaths, with more than 80% of fatalities occurring in immunocompromised individuals [1,3]. It is worth noting that *C. neoformans* is characterized by its distinctive polysaccharide capsule [4], which plays a key role in its virulence by protecting the organism from host immune responses. This encapsulated structure primarily inhibits phagocytosis by immune cells such as macrophages, thereby protecting the fungus from being engulfed and destroyed [5]. The fungus also produces melanin, which is a significant virulence factor found in the cell wall and contributes to the organism’s resistance to oxidative stress and antifungal components [6,7]. These, among many other immune evasion strategies, pose challenges for treatment. Flucytosine, fluconazole, and amphotericin B in different combinations are currently the main drugs used for therapy [8]. However, these antifungals have a number of gastrointestinal adverse effects in addition to nephro- and neurotoxicity, which make their administration challenging [9], highlighting the urgent need for alternative antifungal agents with better safety profiles.

Venomous arthropods, such as scorpions and spiders, are rich sources of bioactive molecules, including antimicrobial peptides (AMPs) [10] with significant potential for biomedical and pharmaceutical applications [11]. Spiders are the most abundant terrestrial venomous animals, and, in recent years, their venoms have been characterized for their anticancer, antiviral, antimicrobial, antiparasitic, analgesic, and neurotoxic activities [12]. Studies on the functional and structural diversity of protein toxins present in their venoms have bolstered efforts in antifungal drug discovery. For example, Lycotoxins I and II, the first spider venom-derived AMPs isolated from wolf-spider *Hogna carolinensis*, stand out as neuroactive and antimicrobial peptides showing strong activity on Gram-negative bacteria as well as fungi [13]. The sheer number of spider species and the challenges associated with venom collection make it difficult to comprehensively study venom components. Consequently, recent studies rely on spider families classified under infraorder Mygalomorphae, whose members are more readily available and produce sufficient venom for analysis [14].

The Mygalomorph spiders of the family Theraphosidae (often referred to as “tarantulas”) are evidently the subject of ongoing research worldwide. The family contains 1117 species classified into 168 genera from all continents, except Antarctica [15]. The main sources of compounds isolated from tarantulas are their venom and hemocytes, both of which have been well studied, with estimates suggesting that 28% of known spider toxin sequences deposited in public databases belong to members of this family [16]. A diverse array of toxins has been reported from theraphosid venoms, such as inhibitory cystine knot (ICK) toxins [17], neurotoxins [18], and zadotoxins [19]. Additionally, gomesin [20], juruin [21], and rondonin [22] are some of the antimicrobial peptides that have been identified from tarantulas.

While most spider venom-derived peptides are isolated and purified to produce functional polypeptides, traditional venom extraction methods are often inefficient and require a substantial number of live spiders, which complicate venom collection and significantly limit the discovery of bioactive compounds. The advent of advanced transcriptomics and structural biology techniques has revolutionized the discovery of AMPs by providing access to vast quantities of biological data and resources. This has greatly enhanced the discovery of toxin diversity in spiders and novel AMPs [23,24].

In this study, a potent antifungal peptide (QS18) with four cysteine residues was identified through in silico analyses of the venom gland transcriptome of the spider *Chilobrachys liboensis*. Linear QS18 with free cysteines showing a reduced state (hereafter referred to as QS18red) demonstrated little to no antifungal activity, prompting us to perform oxidative folding using the GSH/GSSG redox system [25]. The oxidized QS18 constrained by disulfide bonds (hereafter referred to as QS18) exhibited considerable action through rapid membrane disruption, which led us to focus on it in subsequent functional tests. The in vivo systemic infection model suggested that QS18 inhibited the dissemination of fungi to target organs with a controlled inflammatory response, displaying a favorable characteristic as a novel drug candidate.

QS18 addresses the current challenges in treating *C. neoformans* by targeting the fungal membrane, disrupting its integrity and compromising the pathogen’s survival. This membrane damage impairs essential processes such as nutrient transport and cellular signaling, rendering the fungus more susceptible to immune responses. Phylogenetic analysis further supports the potential of QS18 by confirming the presence of tachyplesins in spider venom, which provides valuable templates for the development of peptide antibiotics. In conclusion, QS18 is a promising AMP that warrants further research into its structure–function relationships and spectrum of activity. Its mechanism of action offers a hopeful strategy for combating *C. neoformans* and its robust immune evasion strategies.

## 2. Materials and Methods

### 2.1. Study Design

This study’s research methodology focused on sequencing spider venom glands from which the screening and the identification of spider antimicrobial peptides were conducted. The identified peptides were synthesized through a solid-phase method. A structural analysis of the peptide was conducted, followed by the evaluation of its mechanism of action through in vitro and in vivo assays.

### 2.2. Sample Preparation

One female *C. liboensis* spider species was collected in Tongmian town, Ningming County, Chongzuo City in Guangxi Province, China. The spider chelicerae (fang-bearing appendages) were dissected, and venom glands extracted as described by [26]. The glands were stored in cryotubes under liquid nitrogen for preservation. The liquid nitrogen preserved samples were sent to Novogene Company (Beijing, China) for total RNA extraction and subsequent sequencing. RNA was isolated using a TRIzol reagent (Invitrogen Life Technologies, Carlsbad, CA, USA), and, then, its concentration was detected using a Nanodrop (Wilmington, DE, USA) and Agilent 5400 fragment analyzer (Santa Clara, CA, USA). RNA integrity and quality were checked using the Agilent 5400 system, and purity was confirmed through agarose gel electrophoresis and Agilent 5400. Illumina’s NEBNext UltraTM RNA Library Prep Kit (San Diego, CA, USA) was used to convert RNA samples into a library of cDNA (complementary DNA) fragments for sequencing using the Illumina NovaSeq 6000 sequencing system.

### 2.3. Transcriptome Sequencing

Raw RNA sequencing data were assembled using RNA-Bloom. Transdecoder software (https://github.com/TransDecoder/TransDecoder) was utilized to identify potential protein-coding regions within the RNA data to find potential open reading frames. The possible sequences that code for proteins were identified, and names of the proteins were obtained by Diamond annotations (the reference proteins were obtained from the UniProtKB/Swiss-Prot database). Upon obtaining the protein sequences, their names, functions, and action pathways were further analyzed and annotated using the Pfam library provided by InterProScan (https://www.ebi.ac.uk/interpro/). The resulting matched sequences were compared with entries in the UniProt database using the protein BLAST algorithm (https://www.uniprot.org/blast). Predictions were made using SignalP6.0 (https://services.healthtech.dtu.dk/services/SignalP-6.0/) to identify specific proteins that have signal peptides (short sequences that direct the delivery of the protein to its proper location in the cell).

### 2.4. Sequence Screening and Characterization of Antimicrobial Peptides

The results obtained from the three different bioinformatics analyses—Diamond annotation, InterProScan annotation, and SignalP6.0 prediction—were integrated into a single dataset using the CSV Toolkit. The dataset was filtered to select sequences with 200 amino acids or shorter protein lengths and to possess transmembrane structures as indicated by the SignalP6.0 prediction results. This was performed to obtain the specific protein sequences of interest, which are potential novel antimicrobial peptides.

Based on the software results, along with the number and position of cysteines in the sequences, the scope of the toxin family was approximately determined. To refine the sequences further, comparison to real protein sequences available in the National Center for Biotechnology Information (NCBI) and UniProt databases was performed. Proteins from the toxin family were trimmed based on the closest matches from these databases. Different constituents of the protein, involving signal peptide and precursor regions, were subsequently labeled. Toxin peptides generally contain these regions for secretion and functional maturation.

### 2.5. Peptide Synthesis and Post-Synthesis Modification

For subsequent functional testing, QS18 (QCFKVCFRKRCFTKCSRS) was synthesized using the Fmoc method (purity > 95%, confirmed by Reverse phase high-performance liquid chromatography (RP-HPLC) and mass spectrometry) by Hangzhou Gutuo Biotechnology Co., Ltd., Hangzhou, China.

Additional post-synthesis modification through oxidative folding was performed on QS18 as it comprises cysteine residues in its sequence, a clear indication of possible disulfide bond formation. Oxidative folding was conducted at room temperature for 24 h in a buffer (containing 0.5 mM of GSSG (oxidized glutathione), 0.1 M of Tris-HCL, 5 mM of GSH (reduced glutathione), and 0.1 M of NaCl (at pH 7.4) followed by RP-HPLC purification (with a C18 column measuring 10 mm × 250 mm). The molecular mass of the peptide was confirmed using mass spectrometry. The oxidized QS18 was used in subsequent experiments.

### 2.6. Structural Analysis

Circular dichroism spectroscopy was performed as detailed by [27] to assess QS18 as well as TP1’s secondary structures to understand the peptides’ conformational changes in different environments. QS18 and TP1 (a 2.7 mM final concentration) were detected in phosphate-buffered saline (PBS)-0% trifluoroethanol (TFE), 10 mM of sodium dodecyl-sulfate (SDS), and 40% TFE on a JASCO J-725 spectropolarimeter (Jasco, Inc., Easton, MD, USA) equipped with a quartz cuvette at ambient temperature. The wavelength range for the measured spectra was 190–250 nm with a bandwidth at 1.0 nm and a pathlength of 1 mm at 20 nanometers per minute scanning speed. Each data point was scanned four times under nitrogen gas. The following formula was used to calculate mean residue ellipticity: [θ]M = (θObs × 1000)/(N × L × C) as pointed out by [28], where θObs is the ellipticity observed at a certain wavelength (mdeg), N the number of amino acids, L the pathlength (mm), C the peptide concentration (mM), and θM the mean residue ellipticity (deg cm^2^ dmol^−1^). Besides experimental structural analysis, a computational structure prediction was performed. PEP-FOLD3 was utilized to model the three-dimensional structures of QS18 and TP1.

### 2.7. Antifungal Susceptibility Testing

The minimum inhibitory concentration (MIC) of QS18 against fungi strains was evaluated using the broth microdilution method [26]. To achieve a final cell concentration of 2 × 10^6^ CFU/mL, mid-log phase cultures were diluted with a Yeast and Mold (YM) broth and then put to 96-well plates with two-fold serially diluted peptides (from 0.1 to 89.8 µM) in a 1:1 ratio. Cells treated with amphotericin B or fluconazole served as positive controls, while untreated cells were used as negative controls. After 24 h of incubation at 37 °C, the MIC was determined as the lowest peptide concentration that fully inhibited microbial growth.

Besides the microdilution method, a disk diffusion assay was also performed to determine the sensitivity of test microbes to antimicrobial agents [29]. Fungal cultures were grown as described above and then spread evenly on respective agar plates using sterile cotton swabs. Antimicrobial disks measuring 6 mm and containing 10 mg/mL of each antimicrobial peptide dissolved in normal saline solution were placed on the agar surface, and incubation was conducted at 37 °C for 24 h. The diameter of the zone of inhibition around each AMP-loaded disk was examined and measured. All experiments were conducted in three independent tests.

### 2.8. Stability and Resistance to Proteolytic Degradation

A growth inhibition test and sodium dodecyl sulfate–polyacrylamide gel electrophoresis (SDS–PAGE) analysis were performed to evaluate QS18’s protease resistance as described by [30] in determining its susceptibility to serine proteolytic enzymes. QS18 (1 mg/mL) was incubated with trypsin and chymotrypsin at a ratio of 250:1 (peptide: enzyme) in a digestion buffer (5 mM of CaCl_2_, 50 mM of Tris–HCl, pH 7.4) at 37 °C for 2 h. The mixture was heated for 10 min at 95 °C to halt enzymatic reaction. A solution of 20 µL of the SDS-PAGE loading buffer (5×) was added to 80 µL of the sample, mixed by vortexing and heated for 5 min at 95 °C. SDS-PAGE analysis was performed using Omni-PAGE™Precast HEPES-Tris Gels. LL37 and colistin antibiotics were used as controls.

To ascertain how protease digestion affects QS18’s antimicrobial effect, the peptide was incubated with chymotrypsin and trypsin (250:1) at 37 °C for 2 h. Upon stopping the enzyme reaction by heat treatment for 10 min at 95 °C, 10 µL of the sample was mixed to 90 µL of the diluted *C. neoformans* cell suspension (1 × 10^6^ CFU/mL) in 96-well plates at a final concentration of 4 × MIC QS18 and the positive control, irrespective of whether it was digested or not. Incubation of the mixture was performed for 3 h at 37 °C. A volume of 100 µL of fresh yeast extract peptone dextrose agar (YPDA) broth media was added to the mixture followed by a further 16 h of incubation. Included as negative controls were samples containing heat-inactivated protease with no peptide but media only. The inhibition of fungal growth was detected by measuring absorbance at 595 nm with a Synergy H1 Micro plate Reader (Bio-Tek, Winooski, VT, USA). The assays were performed in three replicates.

### 2.9. In Vitro and In Vivo Toxicity Test

The hemolytic activity of QS18 was determined using human erythrocytes by quantifying the amount of free hemoglobin released from lysis of the cells [29]. Briefly, 100 µL of cells in normal saline solution was incubated with 100 µL of QS18 (0.1 to 45 µM). Following a 30 min incubation period at 37 °C, the cells were centrifuged, and absorbance of the supernatant (hemoglobin) was measured at 540 nm using a microplate reader. Sterile normal saline was used to define “zero hemolysis,” and 1% (*v*/*v*) Triton X-100 was used as the positive control to determine 100% hemolysis. The percentage of Triton X-100-induced hemolysis in the testing sample was used to determine hemolysis. The experiment was performed in technical triplicates.

Cytotoxicity was determined using the HaCaT keratinocyte cells cultured in 96-well plates with Dulbecco’s Modified Eagle’s medium (DMEM/F-12, Gibco, Waltham, MA, USA) containing 1% penicillin–streptomycin and 10% Fetal Bovine Serum (FBS) at 37 °C for 24 h in a humidified 5% CO_2_ atmosphere. After 24 h, fresh media containing peptide concentrations from 89.8 µM to 0.7 µM was added into respective wells in six replicates. Wells containing cells and F-12 media were used as the negative control, wells with F-12 media only used as blank and dimethyl sulfoxide (DMSO) were used as the positive control, and incubation was performed for another 24 h under similar conditions. After 24 h, cell viability was measured by the Cell Counting Kit-8 (CCK-8) assay kit by adding 10 µL of CCK-8 into each well and incubated at 37 °C for 2 h, and, then, absorbance of the colored solution was measured at 450 nm. The color intensity is directly proportional to the number of viable cells in the culture.

An in vivo acute toxicity test was conducted as outlined by [31] to determine the immediate toxicity of QS18 after a single/short-term exposure. C57BL/6J male mice of 6–8 weeks old weighing 20 ± 2 g were injected intravenously or intraperitoneally (n = 6) with a single dose of QS18 at 10 mg/kg, 15 mg/kg, 20 mg/kg, 25 mg/kg, 30 mg/kg, and 40 mg/kg. The peptide was administered in decreasing dosages to the different groups of mice. The concentration was lowered after three repeats if death was immediate, and 6 mice were injected if no death occurred. Following injection, mice were examined visually for any indications of toxicity, and the times at which mice died were recorded at 12/24/48/60 and 72 h. The following signs were used to assess toxicity: hunched back, ruffled fur, poor motility, and total immobility resulting in death.

### 2.10. Time-Killing Kinetics Assay

Time-kill kinetics was carried out as outlined by [24] to assess the fungicidal and fungistatic activity of QS18 over time. *C. neoformans* fungi grown to mid-log phase was adjusted with normal saline to 1 × 10^5^ CFU/mL. QS18 and the positive control (fluconazole) were adjusted to specific concentrations (1/5/10× MIC) using the fungal suspension, and incubation was performed at 37 °C for 0/15/30/60/120/240 min. A volume of 10 µL of the sample was taken at each time point and diluted with sterile normal saline to 100-fold. A total of 100 µL of the dilution was seeded on YPDA media agar plates in three replicates for each concentration per time point. Plates were incubated for 18–24 h at 37 °C, and the number of viable colonies was determined to assess the rate of fungi growth.

### 2.11. Electron Microscopy

Transmission electron microscopy (TEM) alongside scanning electron microscopy (SEM) experiments were conducted in reference with previous research [24] to evaluate the mechanism of action and interactions between QS18 and fungal cells. *C. neoformans* were cultured in YPDA broth to mid-log phase and diluted with normal saline (0.9% sodium chloride) to 1 × 10^9^ CFU/mL. The cultures were centrifuged at 3500 rpm for 5 min, and the supernatant was discarded. After three washes with normal saline, the fungal pellets were re-suspended in normal saline. The suspension was then incubated at 37 °C for 2 h with QS18 (1× and 10× MIC) or saline as the negative control group. At 2 h post-incubation, the suspensions were centrifuged at 3500 rpm for 5 min, the supernatant was aspirated, and the pellets were re-suspended/fixed in 1 mL of 2.5% glutaraldehyde. Fixation was performed overnight at 4 °C. Next, the samples were washed in normal saline and dehydrated in a graded series of ethanol (50%, 70%, 90%, and 100%). In the end, the pellets were placed on aluminum stubs, vacuum sputter-coated in gold, and the Hitachi S-3000N (Tokyo, Japan) SEM was used to examine the membrane morphology.

For transmission electron microscopy, the cells were cultured and harvested as described above. After dehydration, cells were embedded and stained for observation under transmission electron microscopy.

### 2.12. Membrane Permeability Test

The 3,3′-Dipropylthiadicarbocyanine iodide [DiSC3(5)] fluorescent dye was used to assess the effects of QS18 on membrane potential [32]. An overnight culture of *C. neoformans* cells was washed thrice and re-suspended in the HEPES buffer (with 5 mM of HEPES, 20 mM of glucose and 0.1 M of KCl, pH 7.5) to achieve a cell density of 10^8^ CFU/mL. The fungal suspension was then incubated with 10 µM of DiSC3(5) for 30 min at 37 °C in the dark. QS18 was mixed with the fungal suspension in fluorescence-specific 96-well plates, at final concentrations of 1×, 2×, 4× and 8× the MIC in three replicates to 200 µL of final volume per well. PBS was added as the negative control and amphotericin B as the positive control. The negative control PBS-treated wells were used to establish baseline fluorescence. Fluorescence intensity was measured and recorded over time for up to 40 min at excitation/emission wavelengths of 622/670 nm. An increase in fluorescence indicated membrane depolarization, which implies the disruption of membrane integrity.

### 2.13. Biofilm Inhibition and Eradication Assays

The effect of QS18 on inhibiting *C. neoformans* biofilm formation was assessed as described by [33] with little modifications. *C. neoformans* BNCC225501 was cultured in YPDA broth media at 37 °C and 180 rpm to the exponential phase. Fungal cells were harvested by centrifugation at 3500 rpm for 5 min followed by washing thrice using PBS. The cells were re-suspended in the same buffer and concentration diluted to 1 × 10^6^ CFU/mL using YPDA broth media. A total of 100 µL of the fungal solution was added into 96-well plates, and 100 µL of QS18 (8 ×, 4 ×, 2 ×, 1 ×, and 0.5 × the MIC) in sterile normal saline (0.9% sodium chloride) was also added into the respective wells in three replicate wells. Concentrations of 2 × and 1 × the MIC of the positive control (fluconazole) and the negative control group (PBS) were added in their respective wells. The plates were incubated for 48 h at 37 °C. Thereafter, plates were washed thrice in PBS and fixed for 10 min with 100 µL of 99% methanol. Methanol was pipetted out, allowing the plates to dry, and then stained for 5 min with 100 µL of 0.1% crystal violet. Excess stain was washed off with PBS then resolubilized in 95% ethanol. Absorbance was recorded at 600 nm to determine the biofilm inhibitory rate of the peptide.

Evaluation of the peptide’s ability to eradicate *C. neoformans* BNCC225501 biofilms was performed by culturing and harvesting the fungal cells as described in the biofilm inhibition assay above. Incubation of the fungal solution (1 × 10^6^ CFU/mL in YPDA media) was conducted in 96-well plates for 48 h at 37 °C. The plates were washed thrice in PBS, and, then, 100 µL of QS18 at 8×, 4×, 2×, 1×, and 0.5 × the MIC was added in three replicates for every concentration. A volume of 100 µL PBS was added as the negative control group, and 2 ×, 1 × the MIC (fluconazole) was added as the positive control. Plates were washed thrice in PBS and fixed for 10 min with 100 µL of 99% methanol. Methanol was pipetted out, allowing the plates to dry, and then stained for 5 min with 100 µL of 0.1% crystal violet. Excess stain was washed off with PBS, and 95% ethanol (100 µL) was added. Using a microplate reader, absorbance was determined at 600 nm for quantification of the percentage of biofilm eradication.

### 2.14. Two-Photon Laser Scanning Microscope (TPLSM)

The effect of QS18 on *C. neoformans* BNCC225501 biofilms was also assessed using the Two-Photon Laser Scanning Microscope as described by [34] with little modifications. Fluorescein diacetate (FDA) and Propidium iodide (PI) dyes were utilized to stain live and dead cells, respectively. The fungi were cultured in YPDA broth media at 37 °C to the mid-logarithmic phase. Fungal cells were centrifuged at 3500 rpm for 5 min and then washed thrice with normal saline (0.9% sodium chloride). The fungal suspension was diluted to 1 × 10^9^ CFU/mL in YPDA broth media, and, then, 500 µL was added to wells in a 24-well plate. Incubation was performed for 24 h at 37 °C; then, wells were washed thrice with PBS. The peptide was diluted to 0.5×, 1×, 2×, 4×, 8 × the MIC with sterile normal saline, and 500 µL was added to the respective wells. Normal saline was added to the negative control group. The 2× MIC (fluconazole and amphotericin B) were added as positive controls and incubated for 24 h at 37 °C. After incubation, the non-adherent cells were washed with PBS, 250 µL (10 µg/mL) of FDA dye (Solarbio, Beijing, China) was added to each well and left in the dark for 20 min, and, then, 250 µL (5 µg/mL) of PI dye (Solarbio, Beijing, China) was added and co-stained in the dark for 10 min. Microscopy observation was performed utilizing the TPLSM (A1MP+, Nikon, Tokyo, Japan) at 555 nm and 488 nm excitations for PI and FDA, respectively.

### 2.15. Evaluation of the Therapeutic Potential of QS18 In Vivo

#### 2.15.1. Mice Infection and Treatment

Mice systemic infection and the treatment model were performed to assess the dissemination of fungi to target organs as described by [35] with little modifications. C57BL/6J female mice 20 ± 2 g were immunocompromised through the intraperitoneal injection of 150 mg/kg cyclophosphamide for four days prior to infection. *C. neoformans* was then cultured in YPDA broth media to mid-log phase. The fungal cells were harvested by centrifugation at 3500 rpm for 5 min and washed three times in normal saline. Fungal pellets were resuspended in the same buffer, and the optical density (OD) value was adjusted to 4 × 10^7^ CFU/mL. A volume of 200 µL of the fungal solution was i.p. injected into immunocompromised mice (n = 4), a total of 8 × 10^6^ colony-forming units (CFUs) injected per mouse. Twelve hours post-intraperitoneal inoculation of the fungi, treatment was administered. QS18 (1, 2, 4 mg/kg), fluconazole (2, 4 mg/kg), and vehicle (normal saline) were i.p. administered. Twelve hours post-treatment, the mice were sacrificed, and blood was collected through retro-orbital bleeding. Organs (lung, liver, kidney, and spleen) were also harvested and divided into two parts. One portion was weighed and placed into a grinding tube with 1 mL of normal saline and then homogenized in a tissue grinder to assess the fungal burden, while fixation of the other portion was performed for the histopathology examination. Following a 5-fold serial dilution in normal saline, 100 µL of the homogenates were seeded on YPDA plates and incubated at 37 °C for 48 h before counting colonies.

#### 2.15.2. Histopathological Examination

Liver, spleen, kidney, and lung sections were fixed in 4% paraformaldehyde, embedded in paraffin and stained with hematoxylin and eosin for histopathological analysis.

#### 2.15.3. Quantification of Cytokine Levels

Blood collected through retro-orbital bleeding was centrifuged for 25 min at 5000 rpm to isolate plasma. Following the manufacturer’s guidelines, standard enzyme-linked immunosorbent assay (ELISA) kits were used to quantify the cytokine levels of tumor necrosis factor-α (TNF-α), interleukin-6 (IL-6), interleukin--1β (IL-1β), and monocyte chemoattractant protein-1 (MCP-1) in the plasma.

### 2.16. Statistical Analysis

Statistical methods were employed to analyze the structural and functional data obtained from this study to derive significant findings. The differences in mean values across groups were calculated, and findings were reported as the mean standard deviation (SD) or the ± standard error of the mean (SEM). Student’s (unpaired) *t*-test and a one-way analysis of variance (ANOVA) were utilized to ascertain statistically significant disparities and test hypotheses. All data were analyzed using GraphPad Prism v8.0 software. Results with *p*-values less than 0.05 are considered statistically significant.

## 3. Results

### 3.1. Identification of AMPs from the Venom Gland Transcriptome of C. liboensis

Investigation of the venom gland transcriptome was conducted through RNA sequencing employing the Illumina Novaseq 6000 platform. Raw reads totaling 45.44 million were sequenced, and the resulting de novo assembly generated 89,572 transcripts using Trinity software (https://github.com/trinityrnaseq/trinityrnaseq/wiki). Library construction was conducted by searching sequences in the NCBI database against known peptides to screen out sequences with a protein length of less than 200 amino acids and a transmembrane structure. Based on homology analysis utilizing the Basic Local Alignment Search Tool (protein BLAST), nine hypothetical antimicrobial peptides were identified (Table S1). Predictions utilizing the random forest (RF) method on AmpGram (http://biongram.biotech.uni.wroc.pI/AmpGram/) revealed three non-AMPs (NAMPs), whereas the support vector machine (SVM) classifier on CAMP3 (http://www.camp3.bicnirrh.res.in./predict/) categorized one NAMP. QS18 was selected from the transcript GC-BN-1-1:979.p1. QS18 showed sequence similarities with gomesin from the spider *Acanthoscurria gomesiana* and tachyplesins from horseshoe crabs, which are well-known antimicrobial peptides [20].

### 3.2. Physicochemical Parameters of AMPs in C. liboensis Venom Gland Transcriptome

Properties of mature peptide regions truncated at regions exhibiting the typical characteristics of AMPs within the putative transcripts were assessed utilizing the ExPASy ProtParam tool (https://web.expasy.org/protparam/). The molecular mass of the peptides ranged from 1973 Da to 2230 Da. TpI indicates the isoelectric point (pI), the pH at which the peptides have a neutral net charge. The hydrophobicity/solubility of the peptides was determined using the grand average of hydropathicity (GRAVY) score index method. Hydrophilicity is indicated by a negative GRAVY score, and a positive GRAVY score shows hydrophobicity. The analysis indicated three hyprophobic peptides with QS18 having the highest net positive charge (+6) (Table 1), implying that it is more likely to interact with the negatively charged microbial membranes leading to effective antimicrobial activity [36]. In addition, the significant alignment with tachyplesins and polyphemusins suggests that QS18 is a potent spider toxin-like AMP, leading to its further structural and functional investigation in this study.

### 3.3. Sequence Alignment and Phylogenetic Analysis of QS18

**Figure 1:**
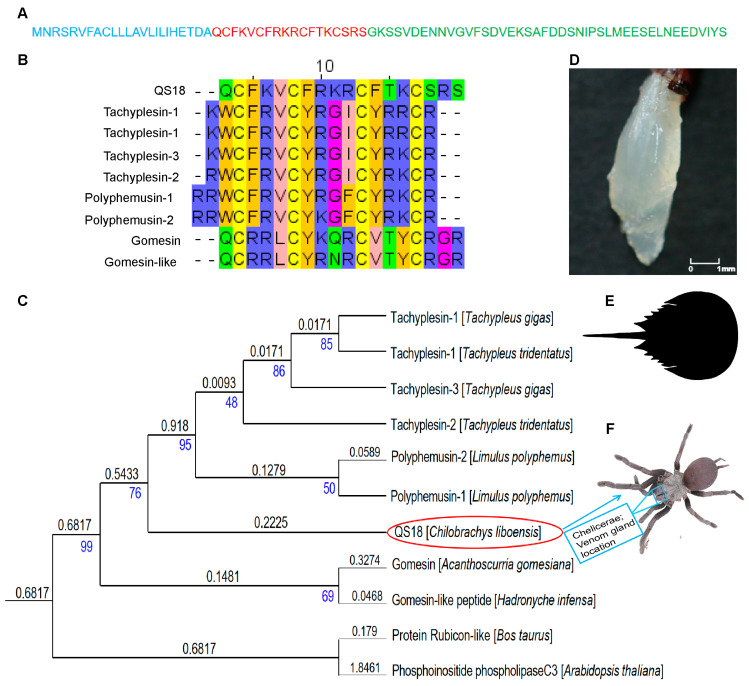
Sequence alignment and phylogenetic analysis for QS18: (**A**) The complete amino acid sequence of QS18. Color-coded regions represent the three typical sections of a toxin peptide sequence: blue for the signal peptide, red for the mature peptide (QS18), and green for the propeptide; (**B**) Sequence alignment utilizing Clustal W software (https://www.genome.jp/tools-bin/clustalw). The amino acid identity is represented in different color codes; (**C**) Phylogenetic analysis maximum likelihood tree generated using RAxML and visualized in the ITOL website. The branch length and bootstrap replications (branch reliability) are represented in black and blue numbers, respectively. The higher the branch length value, the farther the branch. Gomesin (P82358), an antimicrobial peptide from *Acanthoscurria gomesiana* [20]; gomesin-like peptide (A0A1D0BZI2), an antibacterial peptide from *Hadronyche infensa* [17]; Tachyplesin-2 (P14214), from *Tachypleus tridentatus* [39]; Tachyplesin-1 (P14213), from *Tachypleus tridentatus* [39,40]; Tachyplesin-1 (P69135) and Tachyplesin-3 (P18252) from *Tachypleus gigas* [41]; Phosphoinositide phospholipase C 3 (Q56W08), an enzyme involved in signal transduction from *Arabidopsis thaliana* [42]; the protein associated with UVRAG as autophagy enhancer (protein Rubicon-like) (A7E316.1), a regulator of autophagy from Bos taurus; and Polyphemusin-1 (P14215) and Polyphemusin-2 (P14216), AMPs from *Limulus polyphemus* [43]; (**D**) The venom gland of *Chilobrachys liboensis;* (**E**) A silhouette image of a horseshoe crab obtained from PhyloPic (https://www.phylopic.org/); and (**F**) A photograph of the tarantula species *C. liboensis*.

QS18 shared over 50% similarity with tachyplesin and polyphemusin sequences, particularly in the conserved regions (with cysteine (C), phenylalanine (F), and arginine (R) amino acids) (Figure 1B). Tachyplesins are known cysteine-rich AMPs with a β-sheet structural orientation that enhances their broad-spectrum antimicrobial efficacy. For instance, Tachyplesin 1 (TPl) has a wide range of applications, including antibacterial and antifungal properties, and has been previously reported to have membranolytic potential. Additionally, recent studies have reported TP1’s anti-leishmanial properties [37], together with its ability to penetrate cells and cargo delivery in both mammalian and plant cells [38]. The alignment implies that QS18 may have similar structural and functional properties related to antimicrobial activity.

Phylogenetic analysis was performed to assess the relationship between QS18 and tachyplesins (Figure 1C). The results indicated that QS18 is located on the same branch with polyphemusins (with a shorter branch length of 0.1279) and tachyplesins, which possess a shared origin. This close clustering suggests that QS18 has a significant sequence similarity, structural resemblance, or evolutionary relationship to these peptides, which are known for their wide range of antimicrobial properties. QS18 displayed a more distant relationship (0.3274 branch length) with gomesin than the polyphemusin peptides. Protein Rubicon-like and Phosphoinositide phospholipase C3 were included in the tree as distant outgroups that highlight the evolutionary divergence of QS18 and other AMPs from non-AMP proteins.

### 3.4. Synthesis and Purification of Novel Antimicrobial Peptide QS18

Upon synthesis of QS18 by the Fmoc-based solid-phase method and purification using RP-HPLC, mass spectrometry confirmed peptide identity with 98% purity. The synthesized linear QS18 (QS18red) was subjected to an oxidative folding process at 25 °C buffered at pH 7 in the presence of redox reagents GSSG and GSH (1:5). RP-HPLC analysis displayed the main peak of QS18 compound between 36 and 37 min retention time (Figure S2B). The purified compound was collected and lyophilized for further biological assays.

**Figure 2:**
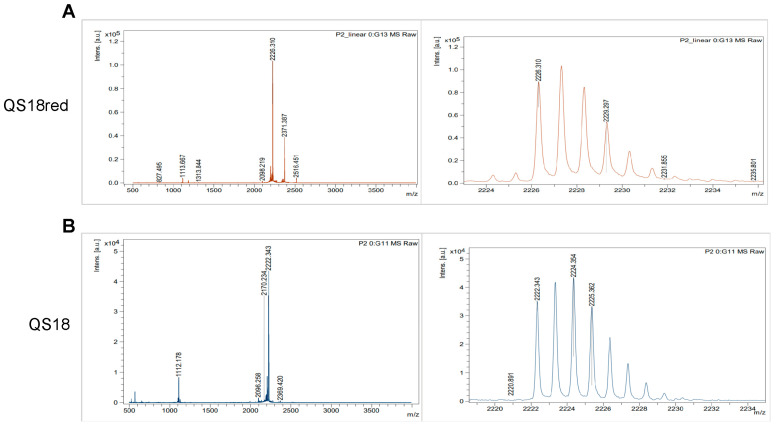
Mass spectrometry analysis: (**A**) Mass spectra of reduced linear QS18 (QS18red). The spectrum shows the mass-to-charge (m/z) ratio distribution, with the highest peak labeled. (**B**) Mass spectra of the oxidized QS18 (QS18). These data confirmed molecular mass and purity of the synthesized QS18 peptide before and after the oxidative folding process. Comparison with the linear peptide spectrum allowed the verification of successful folding and formation of disulfide bonds, as indicated by the decrease in molecular mass.

Mass spectrometry revealed a decrease in molecular mass for QS18 compared to QS18red (Figure 2A), indicating the formation of disulfide bonds and a more compact, correctly folded structure. The mass reduction by 4 Da in the oxidized QS18 (Figure 2B) is in accordance with all final oxidized products’ theoretical masses. The presence of disulfide bonds contributes to stabilizing the native conformation and ensuring the proper functionality of peptides.

### 3.5. Structural Analysis of QS18

**Figure 3:**
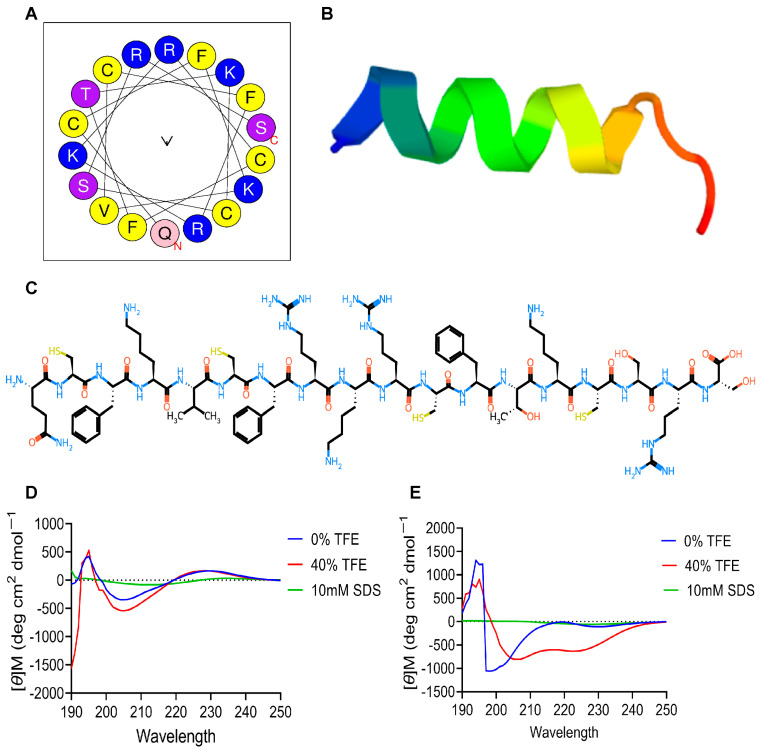
Structural analysis of QS18: (**A**) Helical wheel projections showing QS18’s amphipathic nature. The arrow indicates a hydrophobic moment. Indicated in yellow are the hydrophobic residues, whereas the purple color shows serine and threonine, and blue and pink show the basic residues and glutamine, respectively. C and N are the C- and N-termini represented in red. (**B**) The secondary α-helix structure of QS18 predicted using PEP-FOLD3 software. (**C**) The primary structure of the linear QS18 generated by pepSMI software (https://www.novoprolabs.com/tools/convert-peptide-to-smiles-string). The structure shows amino acids’ sequence connected by peptide bonds. (**D**,**E**) Circular dichroism spectra displaying the secondary helical structure of QS18 and QS18red, respectively, in membrane-mimicking and aqueous solutions: trifluoroethanol (TFE) and sodium dodecyl-sulfate (SDS). [θ]M represents the mean residue ellipticity. Results are expressed as the mean ± standard deviation (SD) of three technical replicates.

QS18’s precursor sequence consists a short 18-mer mature peptide with a 43-mer propeptide region and a 23-mer signal sequence (Figure 1A). The truncated mature segment of the transcript was named QS18, whereby Q represents Glutamine, the first amino acid, S represents Serine, the last amino acid, and 18 represents the total number of amino acids. Sequence analysis of QS18 revealed conserved motifs that are characteristic of antimicrobial peptides. The HeliQuest web server (https://heliquest.ipmc.cnrs.fr) was utilized to further understand the physicochemical properties of QS18, such as hydrophobicity. QS18 adopts an amphipathic form as indicated by the helical wheel projections (Figure 3A). The helical projection demonstrated QS18’s amphipathic nature, with hydrophilic and hydrophobic residues located on opposite sides of the helix. This arrangement is crucial for membrane interaction and antimicrobial function.

Computational secondary structure prediction as per PEP-FOLD3 software via the RPBS Web Portal (https://bioserv.rpbs.univ-paris-diderot.fr) suggested an α-helical structure for QS18 (Figure 3B). Experimental structural analysis confirmed the presence of a more ordered α-helical conformation in QS18 (Figure 3D) than in QS18red (Figure 3E), evidenced by a positive peak at 190–200 nm and a strong negative peak at about 208–222 nm in membrane-mimicking solutions. The spectrum flattened in 10 mM of sodium dodecyl sulfate (SDS) as it neither induces nor stabilizes any secondary structure in peptides.

In this study, we confirmed that, despite the similarity between QS18 and tachyplesins, QS18 assumes an α-helix structure, whereas a representative tachyplesin (TP1) assessed in this study forms a β-hairpin structure in both membrane-mimicking conditions and aqueous solutions (Figure S4). This structural variation emphasizes the diversity within the tachyplesin family and its potential for novel antimicrobial applications.

### 3.6. Evaluation of Toxicity of QS18 to Mammalian Cells

The hemolysis test revealed that QS18 displays a minimal hemolytic effect against mammalian erythrocytes, indicating selectivity towards microbial cells. A percentage hemolysis of less than 10% hemolysis was observed even at high concentrations of 230 µM (512 µg/mL). In contrast, amphotericin-B exhibited over 60% hemolysis at just 8.7 µM (8 µg/mL) (Figure S3A). QS18 displayed negligible toxicity to human keratinocyte (HaCaT) cells, maintaining over 80% cell survival at concentrations up to 45 µM (100 µg/mL) (Figure S3B).

In addition, we performed an in vivo acute toxicity test. QS18 displayed intravenous toxicity, having a lethal effect on mice at 20 mg/kg and 40 mg/kg (Figure S3C). A subsequent dose reduction confirmed QS18’s lethal dose via an intravenous route (LD50 i.v.) at 15 mg/kg, with no lethal effect observed. A toxicity test via intraperitoneal administration confirmed its lethal dose via an intraperitoneal route (LD50 i.p.) at 30 mg/kg, which is two-fold higher compared to intravenous administration.

### 3.7. Protease Resistance

**Figure 4:**
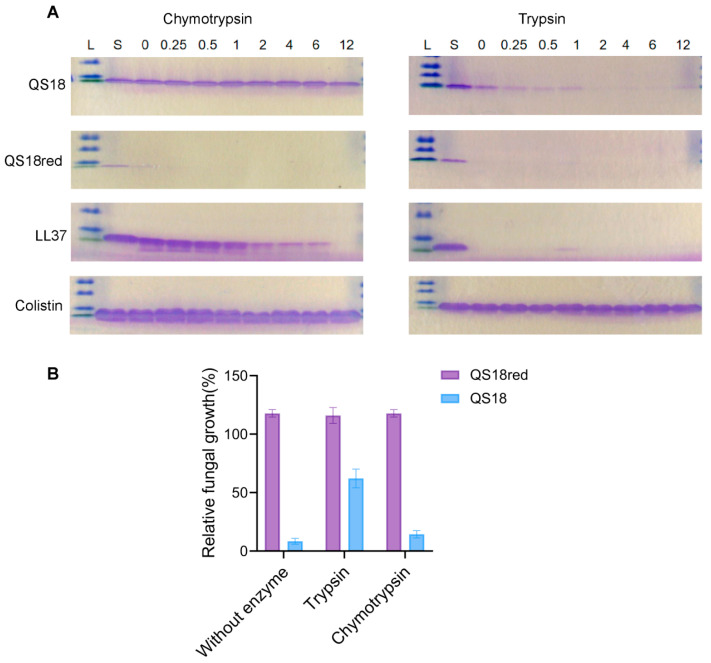
Proteolytic resistance: (**A**) SDS-PAGE evaluation of the proteolytic stability of QS18, QS18red, LL37, and colistin following incubation with chymotrypsin or trypsin from 0 to 12 h. Besides colistin, LL37 human cathelicidin AMP served as a positive control due to its known susceptibility to serine proteases. L represents the protein ladder, and S represents peptide samples without enzymes. (**B**) The effect of proteolytic enzymes on the antifungal effect of QS18. Two hours post-incubation, QS18 appeared to maintain its antimicrobial property in chymotrypsin, which corresponds with the SDS-PAGE analysis results, displaying resistance even up to 12 h. Data represent the mean ± SD of three independent experiments, each performed in technical triplicates.

The inactivation of AMPs by endogenous human proteases and proteases secreted from invasive microorganisms is a significant constraint on their clinical use [30]. A protease degradation assay was performed at different time points up to 12 h to determine the resistance or susceptibility of QS18 to serine proteolytic enzymes. We compared the protease susceptibility of QS18 and QS18red using trypsin and chymotrypsin (peptide: enzyme ratio = 250:1). QS18red showed high susceptibility to proteolysis with trypsin, having no bands observed even at t = 0 min, immediately after incubation with the enzymes. With chymotrypsin, QS18red showed only a faint initial band that disappeared after 15 min of incubation (Figure 4A). In contrast, QS18 demonstrated better protease resistance. It remained stable in chymotrypsin even after 12 h of incubation, showing similar resistance to the control antibiotic colistin. However, QS18 was more susceptible to trypsin, showing complete degradation after 1 h. In addition to colistin, LL37 was used as the positive control. LL37 is a cathelicidin-derived antimicrobial peptide found in humans, and it is known for its broad-spectrum antimicrobial properties and immunomodulatory functions. However, its susceptibility to proteolytic degradation limits its clinical applicability [44].

In order to determine how proteases impact the antifungal action of QS18, we measured *C. neoformans* growth inhibition in the presence and absence of trypsin or chymotrypsin. After 2 h of protease exposure, QS18 treated with trypsin showed reduced antifungal efficacy, evidenced by increased fungal growth. Conversely, QS18 maintained its activity in chymotrypsin (Figure 4B).

### 3.8. Antifungal Activity of QS18

The antifungal property of QS18 was evaluated involving standard and clinically isolated fungi strains. The peptide displayed stronger activity against *C. neoformans* strains. The minimum inhibitory concentration (MIC) test showed that QS18 had the least values of 1.4 µM and 2.8 µM against *C. neoformans* BNCC225501 and *C. neoformans* ATCC32045, respectively (Table S2). According to a comparison of their MIC values, the peptide’s effect was better than that of traditional antifungals like amphotericin B and fluconazole, which are commonly used to treat *C. neoformans* infections. QS18red exhibited little to no antifungal activity. Therefore, given that QS18 manifested considerable ability, we utilized it in the following functional experiments focusing on one fungi strain, *C. neoformans* BNCC225501.

**Figure 5:**
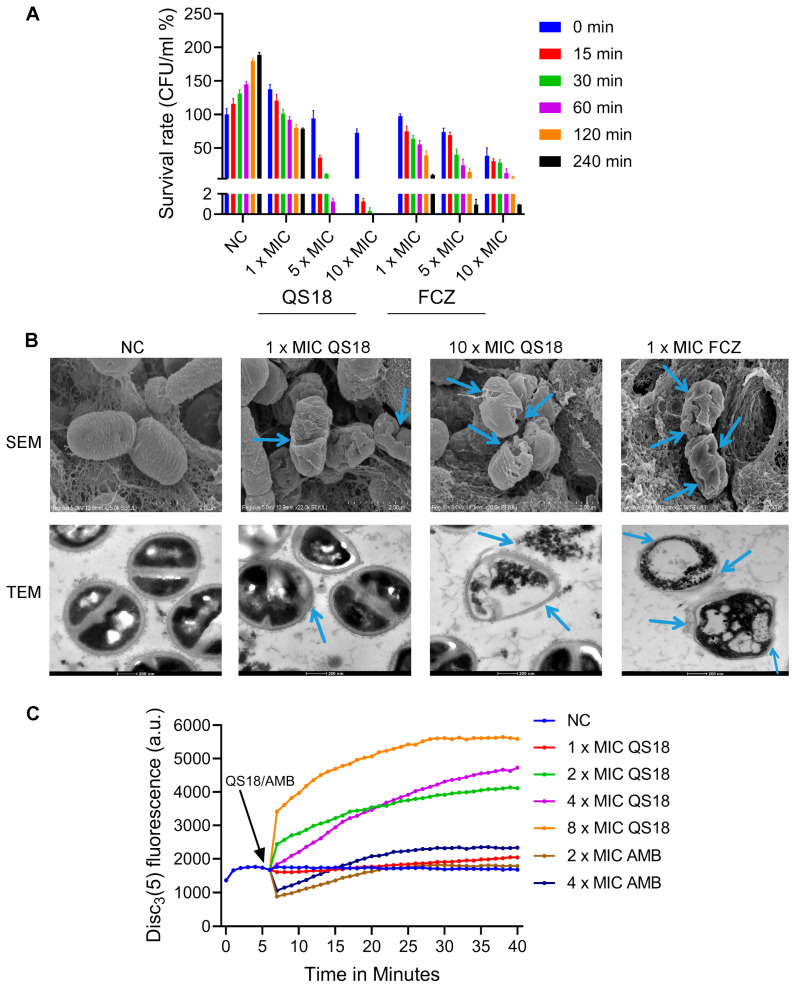
Antifungal activity of QS18 against *C. neoformans* BNCC225501 cells: (**A**) Time-dependent fungicidal activity. QS18 exhibits potent effects with complete growth inhibition at 10× the MIC within 60 min and at 5× the MIC after 120 min. (**B**) Scanning electron microscopy (SEM) and transmission electron microscopy (TEM) micrographs showing the effect of QS18 treatment at 1× and 10× the MIC. SEM and TEM scale bars are 2 µm and 200 µm, respectively. The arrows highlight key areas of membrane disruption, including visible perforations, thus providing visual evidence of QS18’s membrane-targeting mechanism. (**C**) The fluorescence intensity of DiSC3(5) in *C. neoformans* cell suspension. Abbreviations: FCZ represents fluconazole, and AMB represents amphotericin B. The results are expressed as the mean ± SD of three independent experiments, each conducted in technical triplicates.

Further, an assessment of QS18’s action on the viability of *C. neoformans* BNCC225501 fungi over time was conducted to provide insights into its fungicidal (killing) or fungistatic (growth-inhibiting) effects. The peptide displayed substantial fungicidal activity with no fungal growth at 10× the MIC and 5× the MIC after 60 min and 120 min, respectively (Figure 5A). Its rapid action underscores its ability as a therapeutic candidate in treating fungal infections.

Scanning electron microscopy (SEM) and transmission electron microscopy (TEM) were employed in further assessing the peptide’s mode of action. Observations of leaked cytoplasmic contents, as well as irregular cell morphology with shrunken cell surfaces in AMP-treated (10× the MIC; 14 µM) fungal cells, suggest interaction with lipid bilayers, hence, the disruption of the cell membrane integrity (Figure 5B). In contrast, the untreated negative control (NC) cells appeared round or oval with a smooth surface and well-defined borders. Analysis of the SEM and TEM micro-graphs confirmed that QS18’s mechanism of action mainly lies in disintegration of the fungal membrane.

Lastly, a membrane potential measurement assay was conducted using the cationic membrane-permeable fluorescent dye DiSC3(5) to assess the effect of QS18 on cytoplasmic membrane integrity. A significant increase in fluorescence was observed with an increase in peptide concentrations, indicating membrane depolarization (Figure 5C), which leads to cell death.

### 3.9. QS18 Displays Effective Antibiofilm Activity

**Figure 6:**
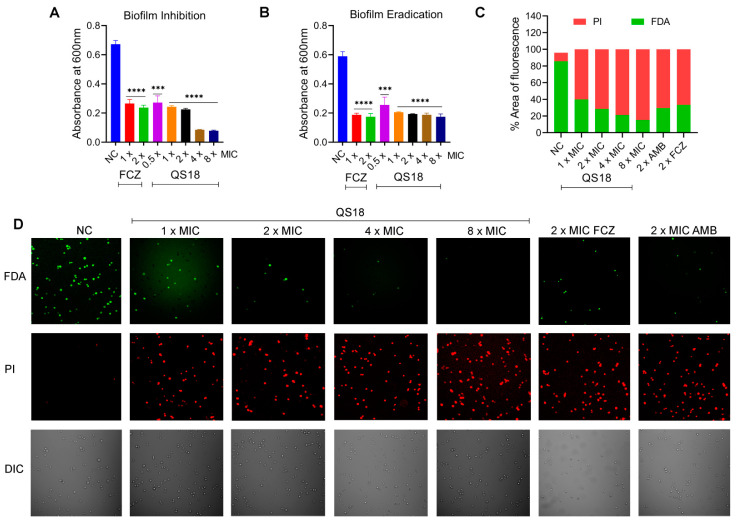
Antibiofilm activity of QS18 against *C. neoformans* BNCC225501 cells: (**A**) Dose–response inhibition of biofilm formation in 96-well plates. (**B**) Dose–response eradication of preformed biofilms in 96-well plates. Data expressed as mean OD600 values of solubilized crystal violet dye ± SEM. (**C**) Quantification of the red (PI) and green (FDA) fluorescence. The percentage area of fluorescence is expressed relative to the negative control (NC). (**D**) Two-photon laser microscopy analysis of the peptide’s effect on *C. neoformans* biofilms: Propidium iodide (PI) and Fluorescein diacetate (FDA) dyes were used to stain the dead and live cells, respectively. Imaging of the differential interference contrast (DIC) under white light was also assessed. Abbreviations: FCZ, fluconazole; and AMB, amphotericin B. Treated *C. neoformans* populations demonstrated a higher proportion of dead cells (red fluorescence) compared to untreated cells (green fluorescence). This color intensity shift indicates significant antibiofilm activity in the presence of QS18. These observations further corroborate the membrane-targeting mechanism of QS18. Scale bar, 50 µm. The data are presented as the mean ± SEM of three independent experiments. A one-way ANOVA was conducted for statistical analysis. Statistical significance: *** *p* < 0.001, and **** *p* < 0.0001.

The increased resistance of biofilms to antifungal treatments, in contrast to planktonic cells, holds considerable clinical significance [45]. For instance, biofilms of *Cryptococcus* spp. display resistance to azole antifungals, whereas amphotericin B and its formulations exhibit great efficacy against biofilms, but their effective dosages exceed the therapeutic range [1]. This results in severe toxicity and renal dysfunction, hence, the development of drug-resistant strains. We evaluated the ability of QS18 to inhibit and eradicate fungal biofilms in vitro. QS18 indicated dose-dependent antibiofilm ability. The peptide markedly inhibited the formation of *C. neoformans* biofilms with 60% inhibition at sub-MIC levels (Figure 6A) and effectively eradicated the preformed biofilms to up to 65% at 1 × MIC (Figure 6B).

Moreover, the effect of QS18 on biofilms was assessed using two-photon laser scanning microscopy. The viability of *C. neoformans* cells decreased dose-dependently, as evidenced by the increased red fluorescence intensity (P1-labeled dead/damaged cells) and the reduction in green fluorescence intensity (FDA-labeled live cells). The negative control (NC) group showed a higher intensity of green fluorescence, suggesting that peptide treatment induced fungal cell damage, resulting in a reduction in green fluorescence and an increase in red fluorescence (Figure 6C,D). QS18 exhibited greater efficacy at as low as 1 × the MIC, comparable to known antifungals used in this study; fluconazole (FCZ) and amphotericin B (AMB). This presents QS18 as a potent candidate in the development of therapeutics.

### 3.10. QS18 Suppresses the Dissemination of Fungi to Target Organs

**Figure 7:**
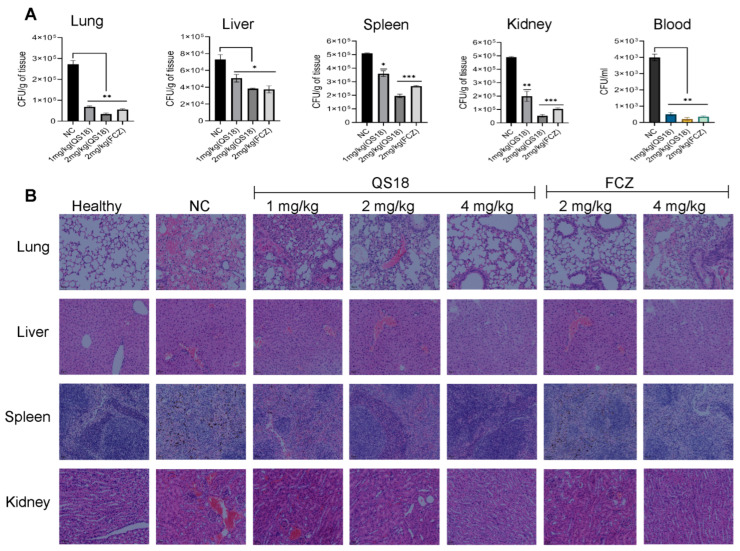
QS18 treatment of mice infected with *C. neoformans*: (**A**) Evaluation of the number of colony-forming units (CFUs) (*C. neoformans*-8 × 10^6^ CFU/mouse, n = 4) per gram of tissue and per mL in blood. Results demonstrate a substantial dose-dependent decrease in fungal burden in treated groups across all examined tissues and blood. (**B**) Histopathological analysis of inflammatory responses. Tissue sections from the lung, liver, spleen and kidney were subjected to hematoxylin and eosin (H&E) staining. Compared to untreated negative controls (NC), QS18-treated sections exhibited dose-dependent reduction in infiltrating inflammatory cells and overall architectural disruption. The 4 mg/kg QS18-treated group shows near-normal tissue morphology, indicating the significant alleviation of infection-induced inflammation. Scale bar, 50 µm. Results are expressed as the mean ± SD of four biological replicates per dose group. A one-way ANOVA was performed. Statistical significance: * *p* < 0.05, ** *p* < 0.01 and *** *p* < 0.001.

*C. neoformans*-induced in vivo systemic infection, and the treatment model was conducted by evaluating the therapeutic ability of QS18. At 12 h post-treatment, various tissues were analyzed for the colony-forming unit (CFU) count (y-axis). As shown in Figure 7A, QS18 demonstrated significant suppression of fungi dissemination to target organs evidenced by the notable reduction in fungal burden (CFU per organ) after peptide treatment. Both the peptide and positive control exhibited dose-dependent fungal suppression. At 2 mg/kg, QS18 displayed an effective in vivo antifungal effect comparable to the positive control’s activity.

Histopathology analysis results revealed that treated groups showed signs of improved tissue health and a more controlled inflammatory response against *C. neoformans* as compared to untreated groups. Observations were made on tissue damage, characterized by widespread necrosis, dilated capillaries filled with red blood cells, and the infiltration of inflammatory cells (predominantly neutrophils) in the NC group, an indicator of fungal infection. There was a decrease in necrosis with fewer infiltrating neutrophils in comparison to the 2/4 mg/kg treated groups, revealing that QS18 treatment was effective in managing the fungal infection (Figure 7B). Consequently, fungal suppression was primarily attributed to the administration of the peptide.

**Figure 8:**
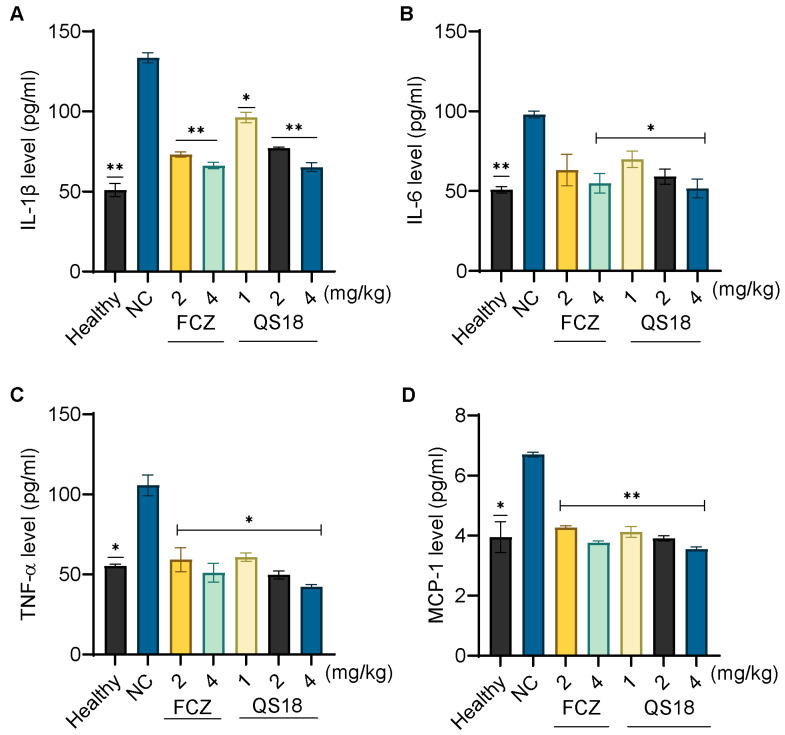
Quantification of pro-inflammatory cytokines: (**A**–**D**) Plasma was analyzed for concentrations of interleukin--1β (IL-1β), interleukin-6 (IL-6), tumor necrosis factor-α (TNF-α), and monocyte chemoattractant protein-1 (MCP-1), respectively, using the enzyme-linked immunosorbent assay (ELISA). QS18-treated groups demonstrated dose-dependent reductions in these inflammatory markers compared to the untreated group. Data represent the mean ± SD of four biological replicates per dose group. Statistical significance was assessed using one-way ANOVA; * *p* < 0.05 and ** *p* < 0.01.

Additionally, the evaluation of cytokine levels in plasma further confirmed the peptide’s therapeutic effect, as shown in Figure 8. These findings imply QS18’s potential application as a novel antifungal agent.

## 4. Discussion

Spider venoms have evolved into complex pharmacopeias through selective pressures over time. The venom of Theraphosidae spiders display considerable pharmacological potential, with various bioactive compounds identified for their antimicrobial, neurotoxic, antiparasitic, hemolytic, cytotoxic, antitumor, and electrophysiological [46]. Nevertheless, there has been little research conducted on the Theraphosidae species in China. Despite the updated record of twenty-four species across six genera [15], only three species have reports detailing the toxicological characterization of their crude venom: *Ornithoctonus huwena* (syn. *Cyriopagopus schmidti*) [47], *Ornithoctonus hainana* (syn. *Cyriopagopus hainanus*) [48], and *Chilobrachys jingzhao* (syn. *Chilobrachys guangxiensis*) [49]. It is worth noting that research on the above three species was primarily carried out around 10 years ago, presumably due to the ecological environment at that time, which allowed for the collection of a sufficient number of spiders for venom extraction. In recent years, environmental degradation and government ecological protection policies have made it increasingly difficult for scientists to collect enough spiders for venom extraction. However, advancements in transcriptome and in silico analyses hold promise for expanding the scope of Theraphosidae research.

The heightened antimicrobial resistance of fungi to conventional antifungals, along with the emergence of resistant strains, remains a global threat. As a result, antimicrobial peptides are attracting attention as a new source of antifungals due to their ability to target pathogens selectively with fewer side effects. Our study explores and expands the utilization of spider venom-derived peptides as potent antimicrobials against fungi, offering a promising approach to combat fungal infections. We used an in silico approach to identify and characterize a novel antimicrobial peptide (QS18) from the venom gland (Figure 1D) transcriptome of *C. liboensis* (Figure 1F), which exhibited considerable anticryptococcal effects. *C. liboensis*, native to China [50], is approximately 6–9 cm in size, and no reports on the identification of its bioactive compounds have been published to date.

QS18 is an 18-residue peptide, which shows notable similarity to tachyplesins and their derivative families (polyphemusins). Meanwhile, QS18 is the first spider venom-derived peptide classified as tachyplesins. Strikingly, structural analysis displays that QS18 assumes an α-helix structure, whereas tachyplesins form a β-hairpin structure in both lipid-mimicking conditions and aqueous solutions [40,51]. Despite the high sequence similarity, QS18’s structural divergence from classical tachyplesins highlights the plasticity of AMPs across different species and their ability to maintain functional similarity through different structural motifs.

A major finding of this study is that the oxidative folding of QS18 markedly enhances its antimicrobial activity and stability, resulting in an MIC that is approximately fourteen times lower that of fluconazole, which is a standard antifungal agent. QS18 has an even number of cysteines, indicating that the cysteine residues can be paired through oxidation to form disulfide (–S–S–) bonds [52,53]. This pairing is essential for stabilizing the native conformation and ensuring the proper functionality of peptides. We confirmed that oxidized QS18 exhibits a stronger anticryptococcal as well as anticandidal effect than the reduced form (QS18red). In addition, QS18 indicates higher antifungal performance compared to typical antifungals, such as fluconazole in our experiments.

Moreover, our study has demonstrated the stability of QS18 in the presence of proteolytic enzymes as well as its safety profile as a novel antifungal candidate. QS18 is more resistant to proteases than QS18red, remaining stable after prolonged exposure to chymotrypsin. The hemolysis assay confirms QS18’s microbial selectivity, suggesting its suitability as a safe therapeutic agent. Time-killing kinetics assay proved that QS18 exerts a more rapid dose-dependent fungicidal effect over time against *C. neoformans* BNCC 225501. Compared to fluconazole, the peptide is more effective, eliminating nearly all fungi within an hour, whereas fluconazole required four hours to kill all fungi.

By utilizing electron microscopy techniques, we found that the more rapid killing effect of QS18 is likely due to its ability to permeabilize membranes, disrupting cellular homeostasis, and causing cell death. In contrast, fluconazole is a known azole fungistatic agent that interferes with intracellular components leading to the inhibition of sterol biosynthesis [54]. Besides its effective membrane disruption mechanism, the effective action of QS18 on biofilms compared to the known antifungals demonstrates its potential for therapeutic applications, warranting further studies. *C. neoformans*’ high morbidity and mortality are caused by biofilms’ resistance to host defense systems and antifungal medications. The ability of QS18 to inhibit and eradicate biofilms to over 50% at sub-MIC levels presents its effective antibiofilm activity on the fungi.

Subsequently, the evaluation of QS18’s efficacy on mice infected with *C. neoformans* revealed a significant dose-dependent reduction in the fungal load. Fungi inoculation and peptide administration was performed intraperitoneally, whereby the lowest and highest dosages administered were fifteen times and about four times lower than the LD50 (i.p.), respectively. There was a substantial decrease in fungal spread to target organs, even at the lowest dosage. Additionally, we conducted an immunomodulatory function assay, analyzing the cytokine concentrations in the plasma of mice infected with *C. neoformans*, and subsequently treated with the QS18 peptide. These pro-inflammatory cytokines activate various types of white blood cells such as neutrophils, which are crucial in the defense mechanism against fungal infections [55]. QS18 manifests a notable regulated immune response, comparable to the fluconazole-treated groups.

Consequently, the findings reported in this study demonstrate QS18 as a novel antifungal peptide. To fully assess the peptide’s efficacy and potential clinical applications, future studies should include broader antifungal screening against more *Cryptococcus neoformans* strains and other clinically relevant fungal pathogens. Furthermore, computational modeling approaches, such as molecular dynamics simulations, are necessary to provide a deeper comprehension of how QS18’s amphipathic α-helix structure correlates with its membrane-targeting mechanism.

## 5. Conclusions

The rising prevalence of drug-resistant pathogens makes the need for novel antimicrobials imperative, and the discovery of QS18 comes at a crucial time in this regard. QS18 is the first bioactive compound identified from *C. liboensis* and the first spider venom-derived AMP identified as a member of the tachyplesin family. The evolutionary relationship between QS18 and tachyplesins is rooted in their structural and functional similarities as antimicrobial peptides derived from different species. The potent antifungal effect and selectivity of QS18 presents it as a prospective candidate for developing novel antimicrobial agents. However, despite the intriguing findings of this study, it is worth noting that the peptide’s toxicity to mammalian cells remains a major limitation in this research, which is a common unfavorable characteristic of many AMPs. Further research into reducing toxicity and optimizing these peptides could lead to significant advancements in antimicrobial therapy, addressing a critical urgency in modern medicine.

## 6. Ethics Approval

Experiments involving animals were approved (IACUC-RE-2024-08-005) by the Institutional Animal Care and Use Committee, Kunming Institute of Zoology, Chinese Academy of Sciences. The C57BL/6J mice (6–8 weeks old) were purchased from Vital River Laboratory Animal Technology Co., Ltd. (Beijing, China).

## Figures and Tables

**Table 1:** Physicochemical parameters of the identified antimicrobial peptides. QS18 is highlighted in red.

Transcript ID	Mature Sequence	Length	MM	TpI	Nc	GRAVY
>GC-BN-1-1:11507.p1	RCRSYCFGKRCLTYCLS	17	2059.47	9.25	4	−0.135
>GC-BN-1-1:19939.p3	YCRSVCGRKRCFTYCKEK	18	2230.67	9.44	5	−0.900
>GC-BN-1-1:25378.p1	QCRSVCISWRCYTYCASS	18	2116.43	8.53	2	0.033
>GC-BN-1-1:36375.p2	QRPDFCKSMRFLKSLKGR	18	2197.65	11.01	5	−1.011
>GC-BN-1-1:5740.p2	QCRSVCISWRCYTYCASS	18	2116.43	8.53	2	0.033
>GC-BN-1-1:78484.p1	QCRSVCFRSRCITYCSS	17	1999.33	8.98	3	−0.041
>GC-BN-1-1:8433.p1	QCRSYCFGKLCLTYCGK	17	1973.37	8.89	3	−0.018
>GC-BN-1-1:979.p1	QCFKVCFRKRCFTKCSRS	18	2227.71	9.94	6	−0.467
>GC-BN-1-1:PDBI077_L03_78483.p4	QCRSVCFRSRCITYCSS	17	1999.33	8.98	3	−0.041

MM: molecular mass; TpI: theoretical pI; Nc: net charge; and GRAVY: grand average of hydropathicity.

## Data Availability

The data supporting the findings of this study are provided in the main text or Supplementary Materials. For further inquiries, the corresponding author can be contacted.

## Supplementary Materials

The following supporting information can be downloaded at https://www.mdpi.com/article/10.3390/microorganisms12122648/s1, Figure S1: RNA library construction; Table S1: Prediction of antimicrobial peptides identified from *Chilobrachys liboensis* spider venom gland transcriptome; Figure S2: Reverse phase high-performance liquid chromatography (RP-HPLC) chromatogram of QS18; Table S2: Minimum inhibitory concentration (MIC) values of QS18 against different fungi strains; Figure S3: In vitro and in vivo toxicity assessment of QS18; and Figure S4: Physicochemical properties of QS18’s tachyplesin analogs.
