# Supplementary material for: A Tachyplesin Antimicrobial Peptide from Theraphosidae Spiders with Potent Antifungal Activity Against Cryptococcus neoformans
Source: Microorganisms. 2024 Dec 20;12(12):2648. doi: 10.3390/microorganisms12122648 (PMC11728142; doi:10.3390/microorganisms12122648)
Supplement: Supplementary file 1 [file microorganisms-12-02648-s001.zip › microorganisms-3348292-supplementary.pdf]

Supplementary Materials

Article

A Tachyplesin Antimicrobial Peptide from Theraphosidae Spiders with Potent Antifungal Activity Against *Cryptococcus neoformans*

Brenda B. Michira[1,2,3,†], Yi Wang[4,†], James Mwangi[1,2,3], Kexin Wang[1,5], Demeke Asmamaw[1,2,3], Dawit Adisu Tadese[1,2,3], Jinai Gao[1,6], Mehwish Khalid[1,2,3], Qiu-Min Lu[1,3], Ren Lai[1,3]*, Juan Li[1,3]*

[1] Key Laboratory of Genetic Evolution & Animal Models, Engineering Laboratory of Peptides of Chinese Academy of Sciences, Key Laboratory of Bioactive Peptides of Yunnan Province, KIZ-CUHK Joint Laboratory of Bioresources and Molecular Research in Common Diseases, National Resource Center for Non-Human Primates, and Sino-African Joint Research Center, New Cornerstone Science Laboratory, Kunming Institute of Zoology, the Chinese Academy of Sciences, Kunming 650201, China.
[2] University of Chinese Academy of Sciences, Beijing, 100049, China.
[3] Kunming College of Life Science, University of Chinese Academy of Sciences, Kunming 650204, China.
[4] Center for Evolution and Conservation Biology, Southern Marine Science and Engineering Guangdong Laboratory (Guangzhou), Guangzhou 511458, China
[5] Medical College of Tianjin University, Tianjin University, Tianjin 300072, China.
[6] School of Molecular Medicine, Hangzhou Institute for Advanced Study, University of Chinese Academy of Sciences, Hangzhou 310024, Zhejiang, China.

†These authors contributed equally to this work.
*Correspondence: rlai@mail.kiz.ac.cn (R.L.); lijuan@mail.kiz.ac.cn (J.L.); Tel.: +86 871 65197578 (R.L.); +86 184 68031104 (J.L.)

A

Summary of test results	
Sample number	FKRO220178262-1A
Nucleic acid number	FZTR220178262-1A
Library type	Eukaryotic common transcriptome library
Concentration (ng/μL)	94.00
Volume (μL)	35.00
Mass (μg)	3.29
Integrity value	8.00
Detection conclusions	Pass
Remarks	Smooth baseline

B

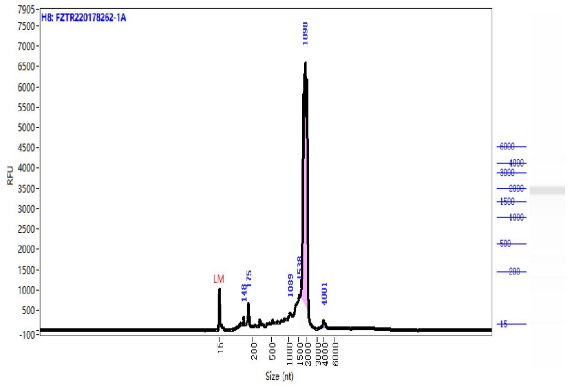

Figure S1: RNA library construction. (**A**) Summary of test results of the RNA sample; (**B**) Analysis of the sample fragment using Agilent 5400 fragment analyzer.

Table S1: Prediction of antimicrobial peptides identified from *Chilobrachys liboensis* spider venom gland transcriptome. The transcript ID highlighted in red represents QS18. Signal, mature and propeptide regions are highlighted in blue, red and green respectively.

Transcript ID	Full-length sequence	CAMPr3 (SVM)	AmpGram (RF)
>GC-BN-1-1:11507.p1	MNRSHVFACLILALLILIHETDARCRSYCFGKRCLTYCLSGSSSADENNVGVFSLVKKSAFDDSNIPSLVEESELNEEGVIFI	AMP 0.758	0.5240
>GC-BN-1-1:19939.p3	MNRTRVFACLFLAVLILIHESDAYCRSVCGRKRCFTYCKEKGVWSSATLVFFRT	NAMP 0.359	0.8376
>GC-BN-1-1:25378.p1	MNRSCVFACLFLAVLILIHGSDAQCRSVCISWRCYTYCASSGKSSVAEKKFGVFSDVEKSDFDDSNIPSLVEESELNDEDAILT	AMP 0.905	0.8138
>GC-BN-1-1:36375.p2	MNRSRVFAFLFLAVLILSLETHAQRPDFCKSMRFLKSLKGRISQIEQMLRSYCESGSSSVDENNVGGFSDVWKSAFDDSNIPSLVEQSELSEEGVIFN	AMP 0.986	0.2732
>GC-BN-1-1:5740.p2	MNRSCVFAFACLFLAVLILIHGSDAQCRSVCISWRCYTYCASSGKSSVAENNVGVFSDVEKSAFDDSNIPSLVEESELNDEDAILT	AMP 0.793	0.5435
>GC-BN-1-1:78484.p1	MNRSRVFACLFLAVLILIHETDAQCRSVCFRSRCITYCSSGKSSVAENNVGVFSDVEKSAFDDSNIPSLVEESELNEEEDVIFS	AMP 0.761	0.3684
>GC-BN-1-	MNRSRVFACLLLIVLICSAVGIDETDAQCRSYCFGKLCLT	AMP	0.5997

1:8433.p1	YCGKGSKSVDENEVGGSSNVTKSAFDDSNNPSLMEEGV SWSSCNFHLSGNSVSIESFSWMKKQKPRKL	1.000	
>GC-BN-1-1:979.p1	MNRSRVFACLLLAVLILIHETDAQCFKVCFRKRCFTKCS RSGKSSVDENNVGVFSDVEKSAFDDSNIPSLMEESELNEE DVIYS	AMP 0.881	0.5782
>GC-BN-1-1:PDBIO77_L03_78483.p4	MNRSRVFACLFLAVLILIHETDAQCRSVCFRSRCITYCSSG KECSWEQRWCFFGRSEKSFRWLQYPIISGSESVK	AMP 0.857	0.4499

Peptides with a probability score above 0.5 are considered likely to have antimicrobial properties

A

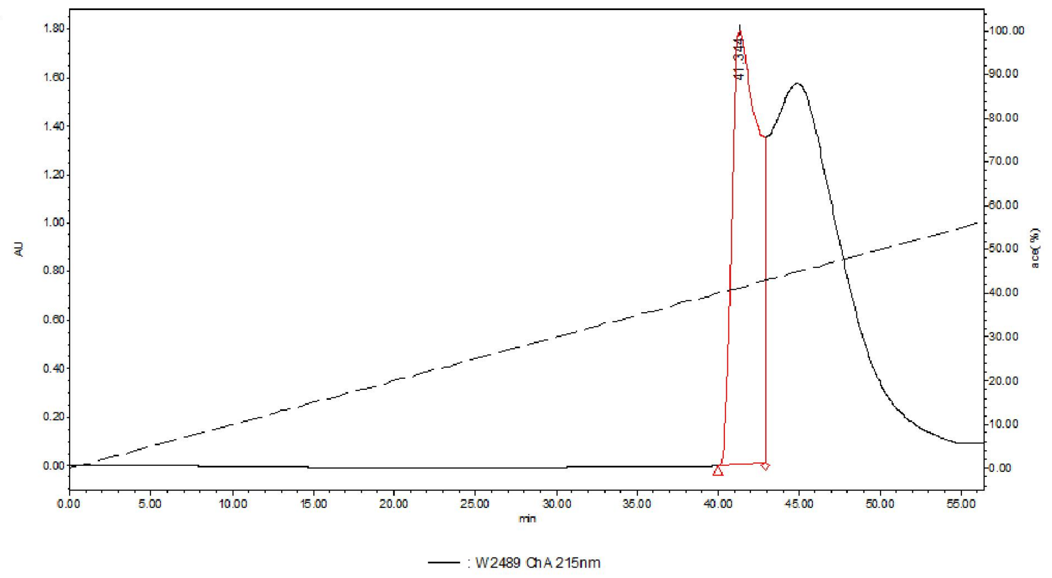

B

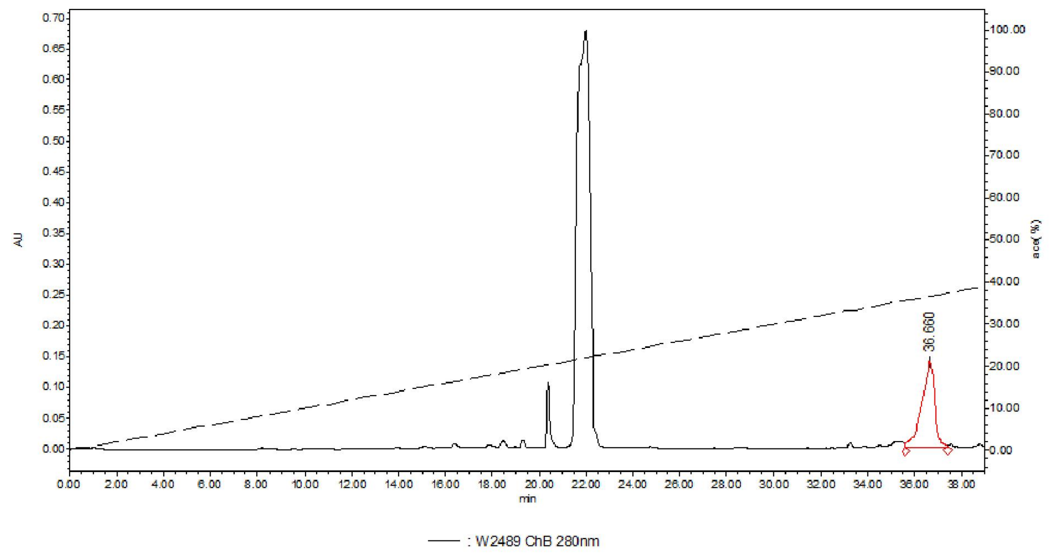

Figure S2: Reverse phase high-performance liquid chromatography (RP-HPLC) chromatogram of QS18. (**A**) QS18red (reduced) eluting at 41 minutes retention time. Absorbance was measured at 215 nm; (**B**)

QS18 (oxidized) eluting at 36.6 minutes retention time. Absorbance was measured at 280 nm. The main peak, representing the QS18 analyte, is highlighted in red. The two earlier peaks observed including a major peak at 22 minutes in the HPLC trace of the oxidized QS18 peptide plausibly correspond to GSH (reduced glutathione) and GSSG (oxidized glutathione), which were used in the peptide oxidation reaction. These two compounds interact with the hydrophobic stationary phase in RP-HPLC hence the observed traces. The oxidized QS18 is more hydrophobic compared to GSH and GSSG. Its hydrophobicity allows it to interact more strongly with the stationary phase, resulting in a longer retention time.

Table S2: Minimum inhibitory concentration (MIC) values of QS18 against different fungi strains

Fungi strain	MIC(μM)			
	QS18red	QS18	Fluconazole	Amphotericin B
*Cryptococcus neoformans*_ATCC32045	≥89.8	2.8	≥40.8	3.4
*Cryptococcus neoformans*_BNCC225501	89.8	1.4	20.4	1.7
*Candida albicans*_ATCC10231	>89.8	≥45.0	≥163.3	≤0.4
*Candida auris*_55	>89.8	22.5	81.6	1.7
*Candida auris*_84	44.9	≥22.5	≤81.6	0.8

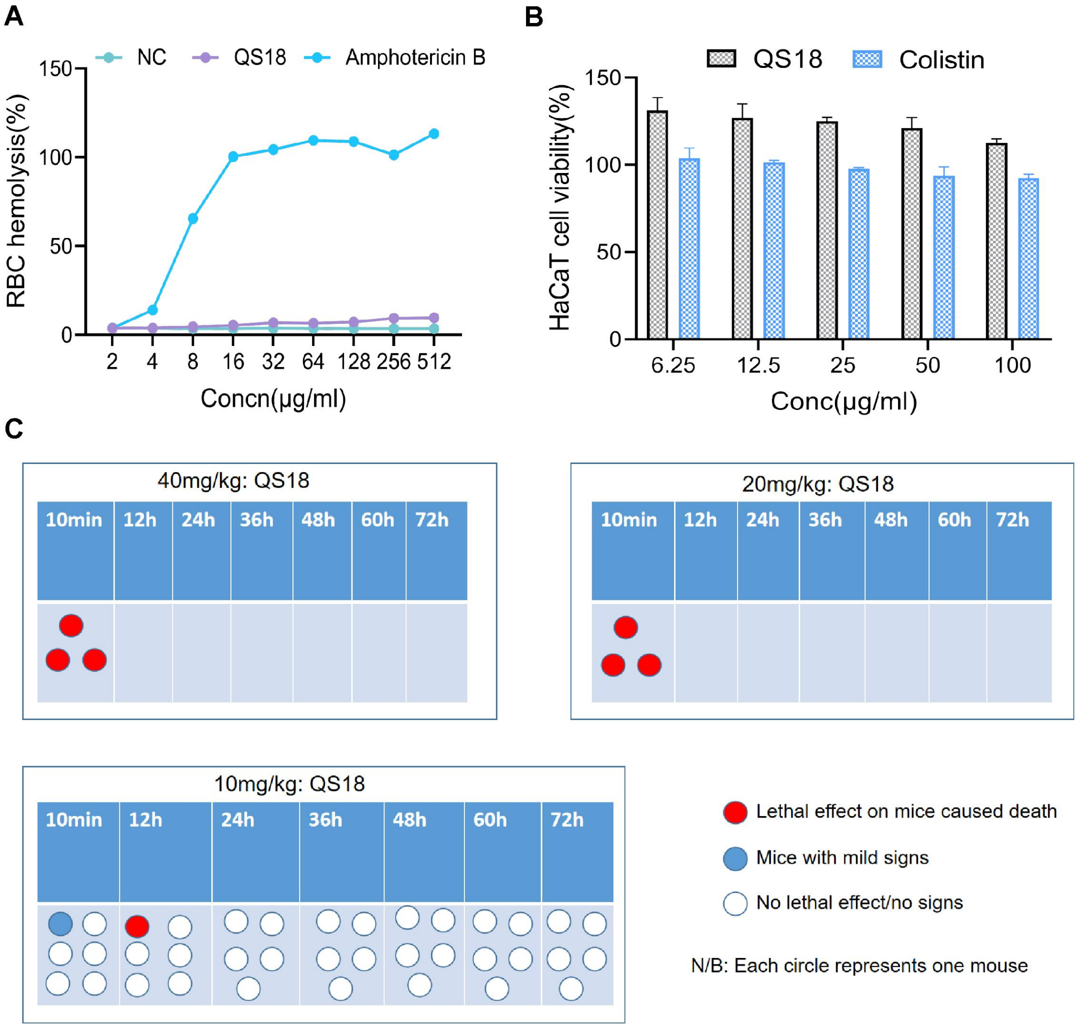

Figure S3: In vitro and in vivo toxicity assessment of QS18. (**A**) Hemolytic activity against human erythrocytes: QS18 demonstrates minimal hemolytic activity, with less than 5% hemolysis observed even at high concentrations of 64 μg/mL (28.8 μM). In contrast, amphotericin-B causes over 60% hemolysis at 8 μg/mL (8.7 μM); (**B**) Effect on human cell viability: QS18 exhibits negligible toxicity on human keratinocyte cells (HaCaT) viability, maintaining over 80% cell survival even at concentrations up to 100 μg/mL (45 μM), indicating low cytotoxicity; (**C**) In vivo acute toxicity in mice: Intravenous administration of QS18 at 40 and 20 mg/kg results in mortality within 10 minutes. However, at 10 mg/kg, no signs of toxicity or death are observed over 72 hours. The legend outlines the toxicity score which is indicated by different circle shading. Data represent the mean ± SD of three technical replicates.

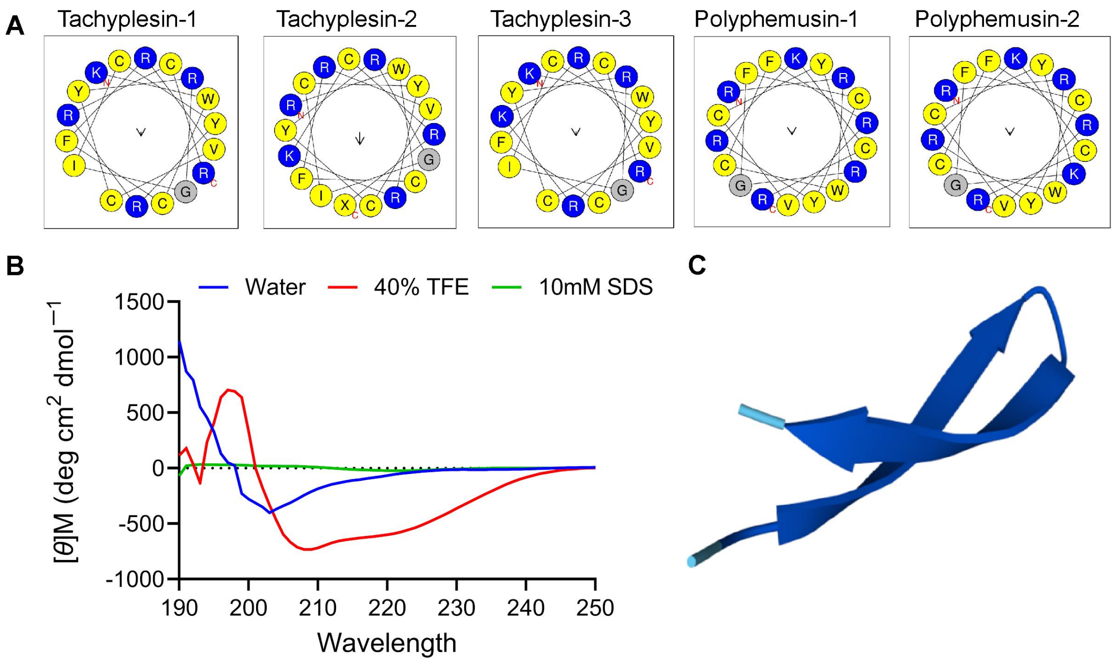

Figure S4: Physicochemical properties of QS18's tachyplesin analogs. (**A**) Helical wheel projections of tachyplesins generated using the HeliQuest web server (https://heliquest.ipmc.cnrs.fr) confirm their amphipathic nature with hydrophilic and hydrophobic residues segregating on opposite sides of the helix. The arrow indicates the hydrophobic moment. Indicated in yellow are the hydrophobic residues, whereas blue indicates the basic residues. C and N are the C- and N-termini represented in red; (**B**) Circular Dichroism spectra of Tachyplesin-1 (TP1) in membrane-mimicking solutions: 10mM sodium dodecyl sulphate (SDS), 0% trifluoroethanol (TFE) and 40% TFE; (**C**) The computational secondary structure prediction as per PEPFOLD 3 software via RPBS Web Portal (https://bioserv.rpbs.univ-paris-diderot.fr) suggests a β-sheet structure for TP1. The model illustrates the secondary beta-sheet structure of tachyplesins. Results are expressed as the mean ± SD of three technical replicates.
